# Patient-Specific Deep Reinforcement Learning for Proton Beam Delivery Under Inter-Phase Variations

**DOI:** 10.1016/j.ijpt.2026.101952

**Published:** 2026-07-22

**Authors:** Mélanie Ghislain, Estelle Loÿen, Antoine Aspeel, Damien Dasnoy-Sumell, Romain Schyns, Ana Maria Barragan Montero, Benoit Macq

**Affiliations:** 1 UCLouvain (ICTEAM), Place du Levant 3, Louvain-la-Neuve, 1348, Belgium; 2 Université Paris-Saclay, CNRS, Gif-sur-Yvette, 91190, France; 3 Multitel Research and Innovation Center (AI Department), Mons, 7000, Belgium; 4 UCLouvain (IREC), Avenue Hippocrate 55, Tour Harvey, Bruxelles, 1200, Belgium

**Keywords:** Reinforcement learning, Mobile tumors, Real-time proton therapy treatment, Plan adaptation

## Abstract

**Purpose:**

Proton therapy is challenged by tumor motion, particularly for lung tumors affected by respiratory-induced motion. Conventional planning strategies compensate for this motion by introducing safety margins or by using robust optimization, increasing irradiation of surrounding healthy tissues. Real-time plan adaptation during delivery represents a promising alternative to mitigate intrafractional motion effects.

**Materials and Methods:**

We propose a patient-specific deep Reinforcement Learning (RL)-based control framework for proton pencil beam scanning, formulated as a first step toward a real-time plan adaptation problem under respiratory motion. RL agents are trained independently on 3 patients on their mid-position planning CT to sequentially control beam position and spot delivery. At inference, the learned policy is executed on the respiratory phases of each patient’s 4DCT without any online re-training or model parameters adaptation. The agent is provided with 2D observations encoding target geometry, beam position, and prior spot delivery. The approach is evaluated against conventional static gross tumor volume-based (GTV-based) and internal target volume-based (ITV-based) planning strategies.

**Results:**

The agents improve target coverage under respiratory motion by exploiting new information in the observations during delivery compared with static gross tumor volume-based plans, with an average gain of 4.54 Gy in $D_{95}^{\mathrm{GTV}}$ over the whole treatment for Patient 1. Compared with ITV-based plans, the RL-based approach generally reduces dose exposure to organ-at-risk, with an average reduction of 0.42, 4.12, and 3.14 Gy in D${}_{\mathrm{mean}}^{\mathrm{Lung-GTV}}$ for the 3 patients, respectively, and a decrease of 1.41 Gy in D${}_{\mathrm{mean}}^{\mathrm{Heart}}$ for Patient 3, who has the largest motion amplitude.

**Conclusion:**

This study should be interpreted as a proof-of-concept and highlights the potential of RL-based control strategies for proton therapy delivery under intrafractional motion. While the current framework relies on static training, it establishes a foundation for future extensions toward fully dynamic and adaptive treatments.

## Introduction

Proton therapy has emerged as a promising modality in radiation oncology due to its ability to deliver highly conformal dose distributions while sparing surrounding healthy tissues. In particular, pencil beam scanning enables the controlled delivery of doses at various depths within the body through energy adjustments. Conventional proton therapy plans are optimized prior to delivery using robust intensity modulation strategies.^1^, ^2^, ^3^ Several delivery techniques have also been proposed to optimize parameters governing spot arrangements at different energy layers.^4^, ^5^, ^6^

However, the treatment of lung tumors poses a challenge for radiotherapy, as it requires adaptation to the continuous motion induced by breathing. This movement can hinder the accurate delivery of radiation doses to the targeted volume. In addition to uncertainties in the proton range, tumor motion during treatment may cause the beam to partially miss the target. Furthermore, when target motion occurs simultaneously with the dynamic scanning of individual proton beam spots, an interplay effect can arise between the temporal pattern of beam delivery and the tumor motion.^7^ This interplay may lead to heterogeneous dose distributions within the target, with potentially larger effects under irregular or large breathing patterns.^8^

To address these challenges, several motion management strategies have been developed. Adaptive radiation therapy (ART) methods rely either on treatment updates before or after fraction delivery or on plan adjustments during treatment fractions using continuous imaging. By incorporating real-time image acquisition, real-time ART enables continuous adaptation of treatment plans to accommodate changes in the patient’s anatomy and physiology throughout the therapy process.^9^ Real-time adjustments can help align the radiation beam more accurately with the tumor, reducing exposure to healthy tissues. These are known as online techniques which account for real-time variations in breathing patterns during a treatment session, such as breath-hold, respiratory gating, and tumor tracking.^10^ In contrast, motion-encompassing approaches such as internal target volume (ITV) construction, abdominal compression, and 4D robust optimization rely on pre-treatment motion modeling rather than real-time adaptation.^11^, ^12^

In real-time ART, the main challenge lies in acquiring accurate anatomical information during treatment and determining an appropriate adaptation strategy based on these observations. To address this need, proton therapy workflows rely on imaging modalities with varying spatial and temporal resolutions, each serving specific purposes in treatment guidance and motion management.^13^, ^14^, ^15^, ^16^ For real-time motion tracking in proton therapy, fluoroscopy, based on continuous X-ray imaging, proves to be a convenient modality due to its non-invasiveness, high temporal resolution, availability, and cost-effectiveness. However, this modality also presents limitations, including reduced soft-tissue contrast, motion blur, and additional radiation exposure.^17^

Machine learning has recently been applied to improve proton therapy planning and robustness evaluation,^18^, ^19^, ^20^ primarily in offline optimization settings. Reinforcement learning (RL) provides a principled framework for sequential decision-making,^21^ while its deep neural network extension, Deep RL (DRL), enables scalable policy approximation in complex, high-dimensional state spaces.^22^ In medical applications, DRL has demonstrated its capacity to address complex sequential decision-making problems.^23^ In radiotherapy, it has been explored for adaptive dose fractionation in non-small cell lung cancer,^24^, ^25^ including knowledge-guided plan optimization,^26^ beam orientation and trajectory optimization for CyberKnife systems.^27^ More recently, RL-based decision support systems have been proposed for response-adaptive radiotherapy, further highlighting the potential of sequential learning frameworks in adaptive oncology workflows.^28^, ^29^

While RL has been explored in the treatment planning stage for objective parameters optimization or spot-weight optimization,^30^, ^31^, ^32^ it has not, to the best of our knowledge, been applied to real-time pencil beam scanning delivery to jointly adapt spot sequencing and spot weights during irradiation. We propose an RL-based delivery controller that selects the next spot location and assigns its delivered weight online from imaging-derived observations, enabling intra-fraction adaptation to anatomical changes. Our method is patient-specific and trained on the Mid-position (MidP) CT and validated on each of the 10 phases of each patient’s 4DCT individually. The results indicate improved Gross Tumor Volume (GTV) coverage relative to a static GTV-based planning strategy, as reflected by increased D${}_{95}^{\mathrm{GTV}}$, suggesting that the RL policy was able to exploit the 2D observations to adapt delivery decisions across respiratory phases. Moreover, the main advantage of the RL-based strategy was observed in comparison with plan_ITV_ for organ-at-risk (OAR)-related metrics. For Patient 2 and Patient 3, lower average OAR doses were observed, suggesting that phase-specific RL decisions may reduce the systematic irradiation associated with the larger ITV volume under intra-fraction anatomical variations, suggesting promising potential for future dynamic training and clinically realistic adaptive proton therapy workflows.

## Materials and method

In this section, we first introduce the proposed RL methodology and then demonstrate how to apply RL to model and tackle ART.

### Reinforcement learning methodology

RL provides a fundamental framework for addressing sequential decision-making scenarios, where an agent aims to find policies maximizing the expected cumulative reward relying on trial-and-error experience, eliminating the need for complete knowledge of the environment a priori.^33^, ^34^

The RL problem is formulated as a discrete-time stochastic control process, involving interactions between an agent and its environment. At each time step, the agent observes the environment, takes an action, receives a reward, and transitions to a new state of the environment. A *Markov Decision Process* (MDP) is a 5-tuple, (*S*, *A*, *T*, *R*, *γ*), where:

• *S* is the state space.
• *A* is the action space.
• *T*: *S* × *A* × *S* → [0, 1] is the transition function, representing conditional transition probabilities.
• R:S×A×S→R is the reward function.
• *γ* ∈ [0, 1) is the discount factor.

MDPs assume full observability, where the observation is identical to the state, facilitating the modeling of agent-environment interactions. Partially Observable Markov Decision Processes (POMDPs) extend traditional MDPs to accommodate uncertainty and partial observability in an agent’s environment. The mathematical formalization of POMDP is stated in.^35^

The *policy*, denoted *π*, is a function from states to actions. It describes how the agent interacts with the environment. The goal of the RL agent is to find a policy that maximizes the expected sum of discounted rewards, ie,

(1) $$J\left(\pi \right)=E\left[\overset{\infty }{\underset{t=0}{\sum }}\gamma^{t}r_{t}\right],$$

where *r*_*t*_ is the reward received at time step *t*. To achieve this, we focus on the *Q-value function*, denoted as *Q*^*π*^(*s*, *a*), which estimates the expected cumulative reward obtained by taking a specific action *a* in a state *s* and then following a particular policy *π* to select the following actions. It is expressed as:

(2) $$Q^{\pi }\left(s,a\right)=E\left[\overset{\infty }{\underset{t=0}{\sum }}\gamma^{t}R\left(s_{t},a_{t},s_{t+1}\right)|s_{0}=s,a_{0}=a,\pi \right].$$

**Figure 1:**
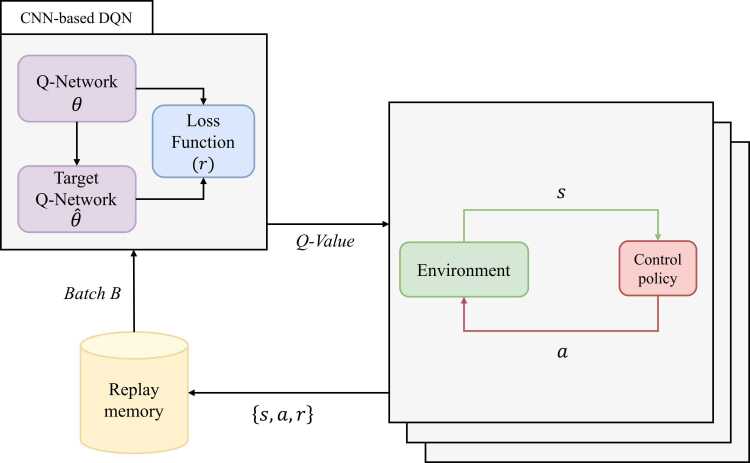
Schematic representation of our RL training loop.

Q-learning is a model-free RL algorithm that iteratively estimates the optimal Q-value function using the Bellman optimality principle and experiences collected from the environment.^33^ In this work, the agent is implemented as a Deep Q-Network (DQN), which uses a neural network and updates its parameters to approximate the Q-value function. The Deep Q-Network loss function (L(θ)) is given by the temporal difference error:

(3) $$L\left(\theta \right)=E\left[{\left(R+\gamma \underset{a_{t+1}}{\max}Q\left(s_{t+1},a_{t+1};\overset{ˆ}{\theta }\right)-Q\left(s_{t},a_{t};\theta \right)\right)}^{2}\right],$$

where *θ* are the online network parameters, and $\overset{ˆ}{\theta }$ are the parameters of a periodically updated target network used for training stabilization. The training method used is presented in Figure 1. The main hyperparameters are summarized in Table S1, and the network architecture is described and illustrated in Figure S2. The algorithm was implemented using the package stable-baselines3.^36^

### Data

Patient data were obtained from Cliniques Universitaires Saint-Luc, Belgium. To evaluate the proposed framework, 3 lung cancer cases with available 4DCT acquisitions are considered. The original CT resolution is 1.17 × 1.17 × 2 mm^3^. The GTV and OAR contours are available for each respiratory phase. The tumors are located in the right upper lobe, right middle lobe, and right lower lobe, respectively, for Patient 1, Patient 2, and Patient 3 (see Figure S1). According to motion amplitude, the peak-to-peak displacement for Patient 1 reached 1.3 mm in the right-left (R-L) direction, 3.5 mm in the anterior-posterior (A-P) direction, and 0.7 mm in the superior-inferior (S-I) direction. For Patient 2, motion amplitudes were 0.9 mm (R-L), 0.5 mm (A-P), and 2.9 mm (S-I), while for Patient 3, 2.1 mm (R-L), 2.5 mm (A-P), and 10.6 mm (S-I).

For all patients, the prescribed dose was 60 Gy delivered in 30 fractions of 2 Gy. Consistent with the GTV-based optimization used in the proposed RL framework, plan_RL_, a GTV-based baseline, plan_GTV_, was generated on the MidP CT using the GTV as the target volume and optimized with the open-source treatment planning system OpenTPS.^37^ Treatment plans were generated independently for each patient using a single proton beam with a fixed couch angle of 0^∘^ and a gantry angle of 270^∘^. A 4 mm margin was applied to the target for spot placement, and both spot spacing and energy layer spacing were set to 4 mm, consistently with previous studies.^38^, ^39^, ^40^ Dose calculations were performed using MCsquare^41^ with a scanner calibration matching the data acquisition setup. Beamlets, used both for reference plan optimization and within the proposed RL-based framework, were precomputed with 10^5^ primary particles per spot, a value aligned with clinical TPS workflows, for which the mean relative statistical uncertainty reported by MCsquare was 5.50%. The final dose calculations were performed using 10^7^ primaries. Reference plan optimization red on the L-BFGS-B solver. For comparison, a motion-encompassing ITV plan, referred to as plan_ITV_, was generated by defining the ITV as the union of the GTV across the 10 respiratory phases and optimizing it on the average CT. All planning, optimization, and dose calculation parameters were kept identical to those of plan_GTV_. The optimized plans doses were subsequently recalculated on each of the 10 respiratory phases of the 4DCT without re-optimization, giving 10 phase-specific dose distributions for plan_GTV_ and 10 for plan_ITV_ for each patient.

### RL environment

**Figure 2:**
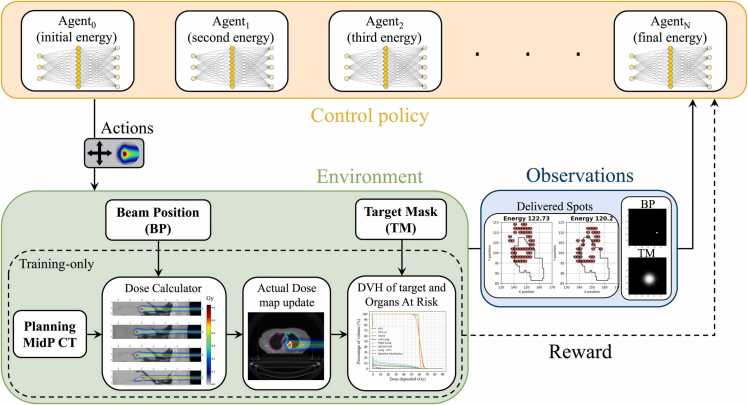
Overview of the multi-energy control framework. The dashed sub-block (“Training-only”) indicates components available only during training (simulating the conditions of real-time delivery).

For the proposed adaptive delivery, proton treatment is formulated as a sequential control problem in which beam displacement, spot placement, and weight modulation are determined during fraction delivery based on 2D anatomical observations. Each episode corresponds to spot delivery at a single energy layer and provides a partial contribution to the prescribed 2 Gy (1 fraction) to the GTV. The beam angle and energy layers used are the same as for the reference plans explained in Section “Data.” The resulting energy layers ${\left\{E_{i}\right\}}_{i=1}^{N}$ are ordered from highest to lowest energy and are attributed *N* independent decision-making agents, 1 agent per energy layer (Figure 2). During training, spot maps are converted into dose increments using MCsquare, enabling computation of the accumulated dose *D*_*t*_ and dose volume histogram (DVH)-based reward terms. This decomposition encourages energy-specific specialization: lower-energy agents learn to complement the spatial irradiation patterns already produced by higher-energy layers while respecting the observability constraints of real clinical workflows. Intuitively, each agent specializes in shaping the spot distribution at a given energy, while conditioning on what has already been delivered at higher energies. The proton beam is characterized by its position (*x*, *y*) in the sagittal plane. The maximum episode length is denoted by $T_{i}\in N$ for energy layer *E*_*i*_. The episode length was determined empirically for each patient and energy layer. An episode may terminate earlier if no spot delivery action (see the section “Actions”) is performed within the initial steps. This prevents uninformative trajectories and improves training efficiency. Full fraction delivery is obtained only after sequential execution of all energy-layer agents. Consequently, an individual episode is not expected to achieve complete target coverage.

### States

The RL agent operates within a 3D environment, where observations are derived from 2D information about the environment, as shown in Figure 2. Since the agent’s observations are given at delivery time, the agent is restricted to 2D clinically plausible matrices:

• *The Beam Position* (BP) is a sparse binary representation where a value of 1 is present at the position (*x*, *y*) of the beam. At the beginning of each episode during training, BP is randomly defined within a 90 × 90 voxel region centered on the target volume. At inference time, BP is initialized using the final BP from the previously optimized energy layer to ensure consistency between successive energy layers.
• *The Target Mask* (TM) in the beam’s eye view is a 2D binary matrix representing the 2D projection of the 3D target mask. In the present study, this observation was generated by projecting the 3D GTV mask from the MidP CT (during training) and from each 4DCT phase (during inference). This simulates a planar imaging acquisition in which a 2D tumor mask is assumed to be available, for instance, from fluoroscopy-based deep learning segmentation or MRI-guided motion monitoring.^13^, ^14^, ^15^

Notably, the SM should help capture an approximation of the dose information essential for assessing dose homogeneity and distribution while the other 2 matrices, BP and TM, assess the spatial aspects of the environment. The problem is formulated as a POMDP, since the agent receives only partial anatomical information through 2D observations. The planning CT and derived dose-based metrics (dose map update, DVH) are treated as *training-only* quantities computed inside the environment to define the loss function but are not accessible to the policy during inference (hence the dashed “training-only” block in the scheme of Figure 2).

### Actions

In this framework, the agent has 2 types of action at its disposal:

• *Beam Movement (BM)* adjusts the position (*x*, *y*) of the beam in 4 directions. Choices include moving the beam up, down, right, or left by 4 mm.
• *Dose Delivery (DD)* administers a spot with a weight of 0.5 MU. This action contributes an incremental dose distribution to the cumulative dose matrix *D*. After the delivery of the selected beamlet, the updated accumulated dose matrix becomes: *D*^(*t*)^ = *D*^(*t*−1)^ + Δ*D*^(*t*)^ where Δ*D*^(*t*)^ is the beamlet dose from the current shot at time *t*.

### Rewards

In this implementation, the reward received by the agent depends on the last action *a* that has been selected; ie,

(4) $$r=\left\{\begin{array}{cc} r^{\mathrm{BM}},\; & a\in \left\{\mathrm{BM}\right\},\\ r^{\mathrm{DD}},\; & a=\mathrm{DD}. \end{array}\right)$$

For a beam movement action, the agent is encouraged to reduce the distance between the current beam position and the GTV, with:

(5) $$r^{\mathrm{BM}}=-\alpha \;d\left(\mathrm{BP},\mathrm{GTV}\right),$$

where *d*(BP, GTV) is the Euclidean distance between the beam position and the target projection in the beam’s eye view, and *α* > 0.

For a dose-delivery action, we evaluate local safety conditions around the Bragg peak region. For simplicity, let *D* denote the current accumulated dose distribution after applying the action at time *t*. The prescribed fraction dose is denoted *D*^⋆^ (2 Gy in our case). Let $N\left(x_{\mathrm{BP}}\right)$ denote a cubic neighborhood extending 6 mm from **x**_BP_ along each Cartesian direction, and let $D_{\max}$ be a safety threshold. In the following equations, *λ*_over_ and *λ*_OAR_ are positive weighting coefficients used to balance the local overdose and OAR protection terms in the reward function. A penalty is applied proportionally to the local overdose:

(6) $$r_{\;\text{local overdose}\;}=-\lambda_{\mathrm{over}}\;\underset{x\in N\left(x_{\mathrm{BP}}\right)}{\max}D\left(x\right)\;\;I\left[\underset{x\in N\left(x_{\mathrm{BP}}\right)}{\max}D\left(x\right)>D_{\max}\right],$$

where I[p] is 1 if the condition *p* holds, 0 otherwise. An additional penalty ensures the protection of OAR:

(7) $$r_{\mathrm{OAR}}=-\lambda_{\mathrm{OAR}}\;\underset{x\in \mathrm{OAR}}{\max}D\left(x\right)\;\;I\left[\underset{x\in \mathrm{OAR}}{\max}D\left(x\right)>D_{\max}^{\mathrm{OAR}}\right].$$

To encourage dose coverage and homogeneity within the target, we use summary statistics over the GTV and OAR. Let D${}_{\max}^{\mathrm{GTV}}$, D${}_{\min}^{\mathrm{GTV}}$, and D${}_{\mathrm{mean}}^{\mathrm{GTV}}$ denote the maximum, minimum, and mean dose within the GTV, respectively. We define a closeness function for a scalar dose statistic z∈R:

(8) $$\phi \left(z\right)=D^{⋆}-|z-D^{⋆}|.$$

The coverage term is defined as:

(9) $$r_{\;\text{GTV cov.}\;}=w_{\max}\;\phi \left(D_{\max}^{\mathrm{GTV}}\right)+w_{\mathrm{mean}}\;\phi \left(D_{\mathrm{mean}}^{\mathrm{GTV}}\right)+w_{\min}\;\phi \left(D_{\min}^{\mathrm{GTV}}\right)\;-w_{\Delta }\;\frac{D_{\max}^{\mathrm{GTV}}-D_{\min}^{\mathrm{GTV}}}{2},$$

where $w_{\max},w_{mean},w_{\min},w_{\Delta }\geq 0$ are weighting coefficients.

Finally, additional penalties are applied to discourage (i) excessive shot density in the same spatial region, with *n*_shot_(**x**) the number of spots already delivered at this position **x** for the considered energy and $n_{\max}$ the corresponding maximum allowed value,

(10) $$r_{\;\text{max weight}}=-\lambda_{\text{max weight}\;}\;I\left[n_{\mathrm{shot}}\left(x\right)>n_{\max}\right],$$

(ii) spot delivery whose Bragg peak falls outside an enlarged GTV margin region, and (iii) any violation of predefined maximum-dose safety thresholds. These 2 additional constraints act as regularization terms that restrict the feasible action space to clinically plausible configurations. By limiting unnecessary exploration of clearly suboptimal or unsafe regions of the state-action space, they contribute to stabilizing and accelerating the training process.

Combining all terms, the dose-delivery reward is given by:

(11) $$r^{\mathrm{DD}}=r_{\;\text{local overdose}\;}+r_{\mathrm{OAR}}+r_{\;\text{max weight}}+r_{\text{GTV cov.}\;}.$$

### Performance evaluation

We compare treatment plans (plan_GTV_ and plan_ITV_) with the phase-specific dose distributions delivered based on the RL agent decision (plan_RL_). Their quality was evaluated using the following dosimetric indicators:

• $D_{\mathrm{mean}}^{V}$: the mean dose received by a specific volume V.
• DVH: a graphical representation showing the proportion of volume receiving a particular dose.
• *Metrics derived from DVH*, such as $D_{a}^{V}$ (dose received by at least *a*% of the specific volume V) and $V_{x}^{V}$ (percentage of volume V receiving at least *x* Gy), are used to assess treatment efficacy and safety.^42^
• *Homogeneity Index* (HI), a measure of the homogeneity of dose distribution within a volume V, 0 representing a total homogeneity. It is computed as $\mathrm{HI}=\frac{D_{2}^{V}-D_{98}^{V}}{D_{50}^{V}}$, see.^43^.

DVHs are generated for GTV, Spinal Cord (SC), Lung-GTV, Heart, and Liver (only for Patient 3) as well as their respective relevant dosimetric indicators. The proposed approach is evaluated as a proof-of-concept to allow assessment of whether the agent trained on a certain anatomy can maintain target coverage during delivery when confronted with an unseen intrafractional change, using only 2D observations to implicitly account for tumor motion.

## Results

**Figure 3:**
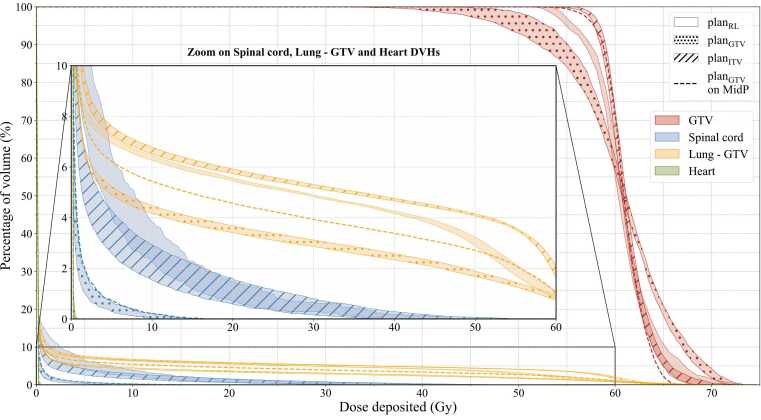
DVH bands of the GTV and OAR for plan_GTV_ (in dotted areas), plan_ITV_ (in dashed zone), and plan_RL_ for Patient 1. Bands denote the phase-wise range over the 10 respiratory phases of the 4DCT. The dashed line corresponds to plan_GTV_ delivered on the MidP CT.

All reported results are presented over the whole treatment (30 fractions of 2 Gy). Figure 3 shows the phase-wise DVHs for Patient 1, both for plan_GTV_, plan_ITV_, and the proposed plan_RL_. The same graph for Patient 2 and Patient 3 can be found in Section “Conclusion,” Figure S3.

**Figure 4:**
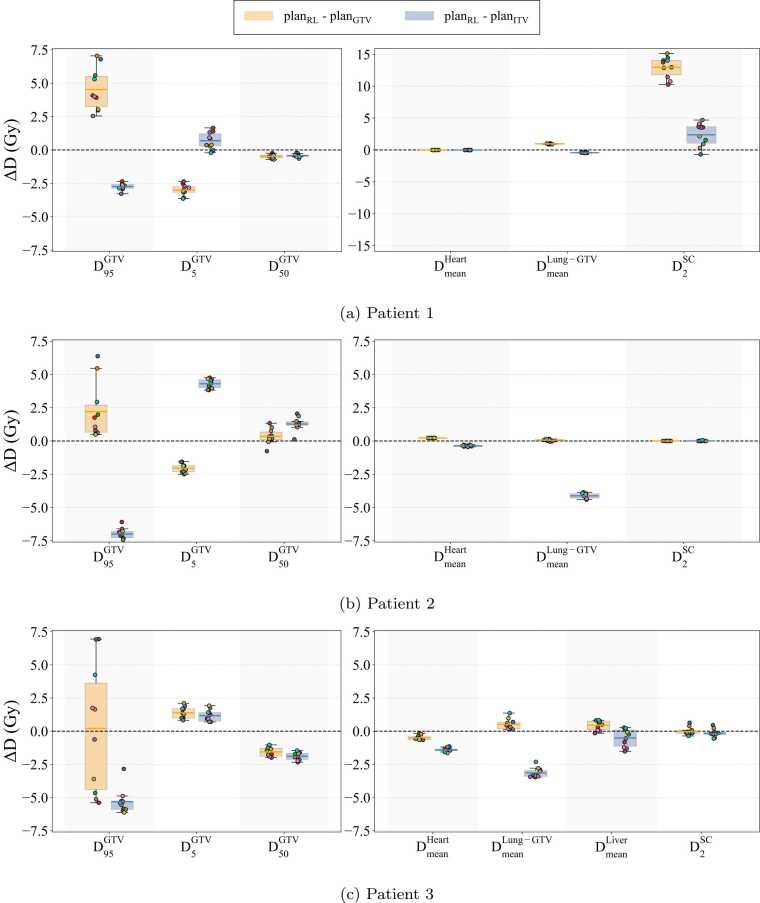
Distribution of phase-wise dosimetric differences between the RL-based approach and 2 reference planning strategies: plan_GTV_ (orange) and plan_ITV_ (blue). Differences are computed after the whole treatment delivery for (a) Patient 1, (b) Patient 2, and (c) Patient 3. Colored dots correspond to individual phases, and horizontal segments indicate mean differences.

Figure 4 reports the phase-wise dose differences between plan_RL_ and both conventional plans for the 3 patients. For target metrics, positive D${}_{95}^{\mathrm{GTV}}$ differences indicate improved coverage of our plan_RL_, whereas for D${}_{5}^{\mathrm{GTV}}$, negative differences mean reduced target high-dose exposure. For OAR metrics, negative differences imply improved sparing with our RL-based approach.

Across the 3 patients, plan_RL_ generally shows higher target coverage than plan_GTV_ as D${}_{95}^{\mathrm{GTV}}$ is increased by 4.54, 2.20, and 0.20 Gy, respectively, while it reduces target high-dose exposure for the 2 first patients. However, differences between plan_RL_ and plan_GTV_ in OAR doses are negligible, except for $D_{2}^{\mathrm{SC}}$ of Patient 1, for which plan_RL_ results in a mean increase of 12.93 Gy.

Conversely, relative to plan_ITV_, plan_RL_ shows a less homogeneous target dose distribution since D${}_{95}^{\mathrm{GTV}}$ is decreased by 2.73, 6.99, and 5.32 Gy for the 3 patients, respectively, and D${}_{5}^{\mathrm{GTV}}$ is increased in several cases. However, plan_RL_ generally reduces OAR exposure compared with plan_ITV_, except for Patient 1, for which $D_{2}^{\mathrm{SC}}$ is increased by 2.36 Gy. For the 2 other patients, plan_RL_ reduces D${}_{2}^{\mathrm{Heart}}$ by 7.48 and 30.01 Gy on average. Overall, plan_RL_ also decreases D${}_{\mathrm{mean}}^{\mathrm{Lung-GTV}}$ by 0.42, 4.12, and 3.13 Gy compared with plan_ITV_ across the 3 patients, respectively.

The absolute OAR dose metrics and their corresponding clinical constraints are reported in Section “Conclusion,” Table S2. Most values are below the clinical limits, except in 2 cases. For Patient 3, plan_RL_ is marginally above the constraint V${}_{20\mathrm{Gy}}^{\mathrm{Lung-GTV}}<30\%$ as the result is 30.46%. For patient 1, 3 breathing phases slightly exceed the limit D${}_{0.001\mathrm{cc}}^{\mathrm{SC}}>45$ Gy.

Overall, these results suggest that plan_RL_ can reduce part of the OAR irradiation associated with the larger ITV volume while maintaining improved target coverage relative to the static plan_GTV_. From a computational perspective, the inference time is compatible with real-time delivery constraints. A single decision step requires approximately 2.6 ms, including observations processing (2 ms) and network forward pass (0.6 ms).

## Discussion

Improvements in target coverage of plan_RL_ compared with plan_GTV_ can be explained by the nature of the learned control strategy. During training on the MidP CT, the agent learns to deliver a spatially coherent dose distribution relative to the precise position of the projected target mask. At inference time, the target projection of a phase from the 4DCT may differ from the one observed during training on the MidP CT. The agent observes this geometric shift and adapts the beam position accordingly, effectively translating its learned delivery pattern to the new target position. This improvement was less pronounced for Patient 3, who exhibited the largest motion amplitude and consequently a greater phase-to-phase variability.

However, this adaptation is primarily driven by GTV-geometry information and does not account for phase-dependent changes in the surrounding anatomy, such as variations in traversed tissues (path-length changes) or deformation of nearby OAR or classical uncertainty scenarios considered in 4D robust proton therapy (eg, range and setup perturbations). Therefore, the adaptation observed in this work arises from geometric compensation rather than multiple scenarios optimization, as reflected by occasional OAR dose increases. This can be seen in Patient 1, for which 3 individual phases slightly exceeded the D${}_{0.001\mathrm{cc}}^{\mathrm{SC}}$ constraint with plan_RL_. The increase in D${}_{2}^{\mathrm{SC}}$ should be interpreted cautiously, since plan_GTV_ provides poor GTV coverage and therefore deposites less dose in nearby regions, including in the SC. For Patient 3, V${}_{20\mathrm{Gy}}^{\mathrm{Lung-GTV}}$ slightly exceeded the clinical constraint. This case also corresponds to the largest motion amplitude, highlighting the limited robustness of a policy trained on a unique anatomical configuration. Incorporating uncertainty scenarios and different intrafractional changes in the training data, thanks to data augmentation,^37^ would represent a natural extension toward fully robust adaptive proton therapy. One limitation of the proposed method lies in the discretization of the spot weights. In this work, the delivered monitor units were restricted to increments of 0.5 MU. The relatively coarse discretization used in our RL formulation may limit achievable dose homogeneity or OAR sparing. This choice was motivated by computational considerations and training stability. Future work will investigate higher discretizations (eg, 0.1 MU or 0.01 MU increments) to better approximate a continuous spot-weight modulation.

In the current formulation, the spot map is initialized as empty at the start of each episode and progressively updated as delivery actions are executed. Another potential improvement would be to take advantage of an existing treatment plan and exploit the prior knowledge available from a clinically optimized plan in the observations set.

The current single-beam configuration inherently limits the achievable spatial modulation of the dose distribution, with only 2D observations available compared to the conventional plan. An extension to multiple gantry angles should increase dose conformality and OAR sparing. A possible contribution would consist of training 1 agent per predefined beam angle for each patient, enabling sequential or alternating beam contributions while maintaining a tractable decision space. Furthermore, broader evaluation across a larger patient cohort, including different lobar tumor locations, different tumor volumes, and motion amplitudes, is required to assess the robustness and generalizability of the proposed strategy.

Another limitation is the idealized type of observation used by the RL agent. Here, we isolated delivery control from imaging and online segmentation by assuming informative 2D tumor-mask observations rather than using acquired images directly. In clinical practice, such observations would need to be generated by an upstream module, such as fluoroscopy-guided tumor segmentation or MRI-guided motion monitoring.^13^, ^14^, ^15^ Its reliability would be critical, as segmentation uncertainty, imaging latency, 2D-3D ambiguity, and image artifacts could affect target localization and, indirectly, the RL decisions. These effects were not modeled here and should be addressed in future imaging-to-control adaptive workflows. Emerging foundation-model-based approaches may help improve this upstream module.^44^, ^45^

## Conclusion

This study presents an intra-fraction RL-based adaptive delivery framework that dynamically adjusts beam position and spot weights during treatment, despite being trained exclusively on a single static anatomical configuration. The results indicate that plan_RL_ can improve target coverage relative to a static plan_GTV_ while reducing part of the OAR irradiation associated with plan_ITV_. Future work should incorporate uncertainty scenarios and dynamic anatomical variations during training, extend the framewok to multiple beam angles, and be validated on a larger patient cohort. Such developments should help our promising framework to pave the way toward adaptive proton therapy workflows capable of compensating intra-fraction motion in real-time.

## Ethics

A local ethics committee approval was obtained for the use of all patient data under the agreement “Learning from the past: the MIRO treatment planning data base” which received the ethical approval by the CEHF (Comité d’Ethique hospitalo-facultaire) (2019/16SEP/402).

## Funding

Mélanie Ghislain and Romain Schyns are funded by F. R. S-FNRS (Fonds de la Recherche Scientifique) under Télévie grants with reference 7.6513.25 and 7.4583.25, respectively. Estelle Loÿen is funded by the MedReSyst project, supported by FEDER and the Walloon Region. Ana M. Barragán Montero is a Research Associate with F. R. S.-FNRS (Fonds de la Recherche Scientifique).

## CRediT authorship contribution statement

**Mélanie Ghislain:** Conceptualization, Methodology, Software, Validation, Formal analysis, Investigation, Writing-original draft preparation, Writing-review and editing, Visualization, Funding acquisition. **Estelle Loÿen:** Methodology, Validation, Writing-review and editing. **Antoine Aspeel:** Methodology, Writing-review and editing. **Damien Dasnoy-Sumell:** Methodology, Writing-review and editing. **Romain Schyns:** Software. **Ana Maria Barragan Montero:** Conceptualization, Methodology, Validation, Writing-review and editing, Supervision. **Benoit Macq:** Conceptualization, Methodology, Validation, Supervision, Funding acquisition. All authors have read and agreed to the published version of the manuscript.

## Declaration of Generative AI and AI-Assisted Technologies in the Manuscript Preparation Process

During the preparation of this work the authors used ChatGPT in order to review and enhance the clarity and grammar of the manuscript. After using this tool/service, the authors reviewed and edited the content as needed and take full responsibility for the content of the published article.

## Declaration of Conflicts of Interest

The authors declare the following financial interests/personal relationships, which may be considered as potential competing interests: Melanie Ghislain reports financial support was provided by the Fund for Scientific Research. Romain Schyns reports financial support was provided by the Fund for Scientific Research. Estelle Loÿen reports financial support was provided by MedReSyst project, supported by FEDER and the Walloon Region. Ana Maria Barragan Montero reports financial support was provided by the Fund for Scientific Research. If there are other authors, they declare that they have no known competing financial interests or personal relationships that could have appeared to influence the work reported in this paper.

## Footnotes

Appendix A Supplementary data associated with this article can be found in the online version at doi:10.1016/j.ijpt.2026.101952.
