# Supplementary material for: Patient-Specific Deep Reinforcement Learning for Proton Beam Delivery Under Inter-Phase Variations
Source: Int J Part Ther. 2026 Jul 22;21:101952. doi: 10.1016/j.ijpt.2026.101952 (PMC13451797; doi:10.1016/j.ijpt.2026.101952)
Supplement: Supplementary file 1 [file mmc1.pdf]

Supplementary materials

Patient vizualisation

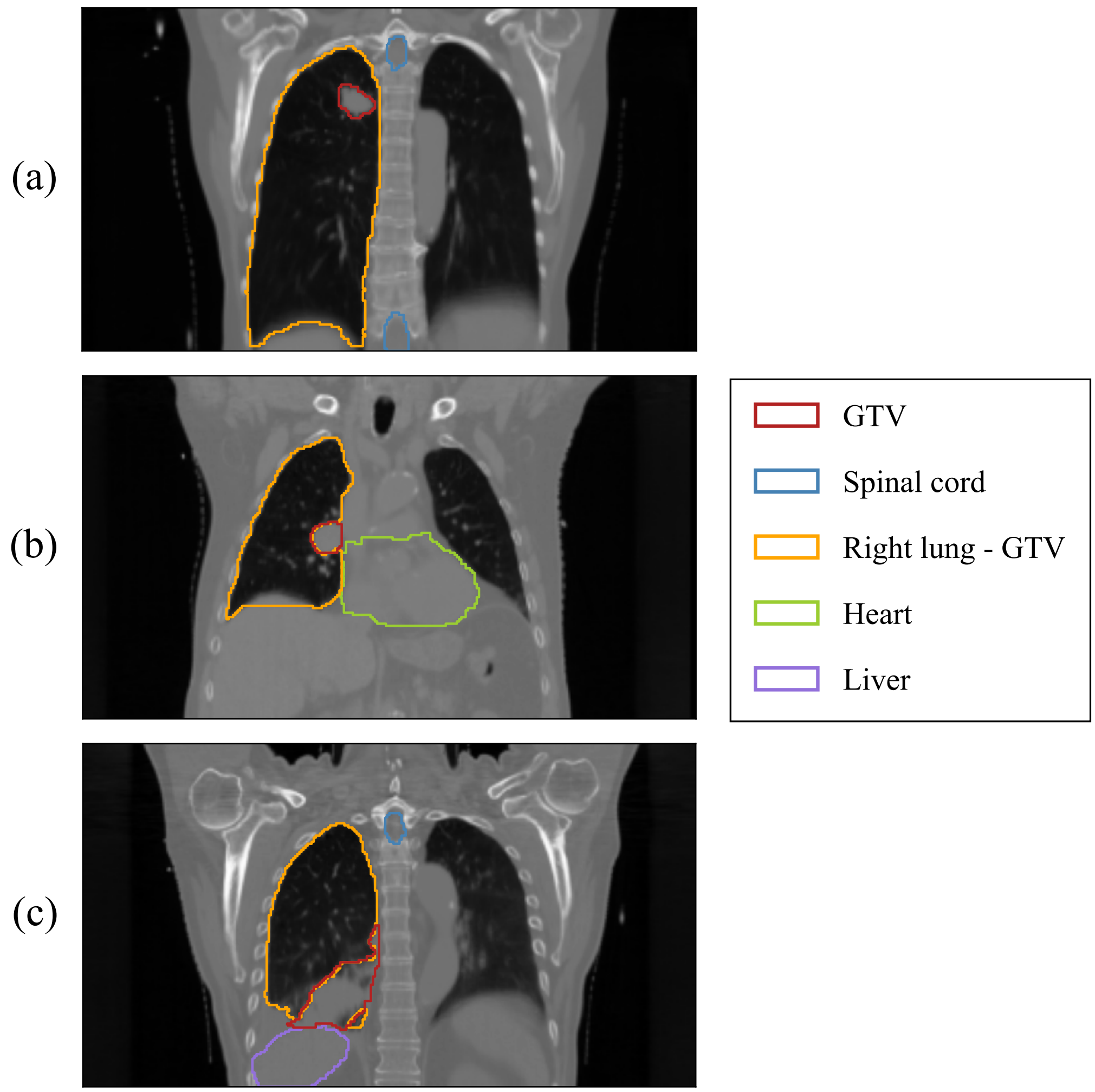

Figure S1: Coronal view of (a) Patient 1, (b) Patient 2 and (c) Patient 3.

Implementation and training details

Training was performed on a single CPU and a fraction of an NVIDIA A100 GPU (80 GB). The training time was approximately 8 hours per patient, including all energy layers.

The agent uses a custom convolutional feature extractor followed by a multi-layer perceptron. The input observation consists of the multi-channel representation. The feature extractor contains three convolutional layers with 16, 32, and 64 filters, respectively, using 3×3 kernels and ReLU activations. A 2×2 max-pooling layer is applied after the first convolution. The resulting feature map is flattened and projected to a 256-dimensional latent representation. This latent vector is then passed to a fully connected network with hidden layers of 128, 64, and 32 units, using ReLU activations. The output layer produces the action values associated with the discrete action space, as shown in Figure S2.

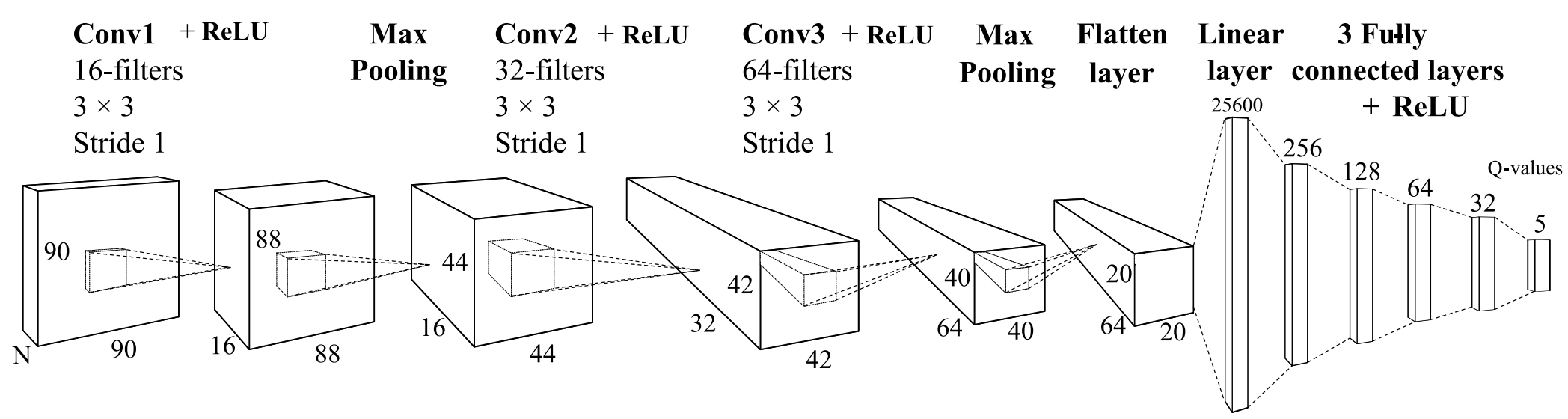

Figure S2: Network architecture.

Table S1: Training hyperparameters of the RL agent

Parameter	Value
Learning rate (α)	0.0001
Discount factor (γ)	0.9
Initial exploration rate (ϵ_0)	0.99
Final exploration rate (ϵ_f)	0.2
Exploration decay rate	0.992
Batch size	16
Steps per episode (T_i)	50-200
Max number of epochs	200
Training episodes per epoch	100
Validation episodes per epoch	30

Clinical OAR constraints and additional results

Table S2: Clinical OAR constraints (for a treatment of 60 Gy in the Lung) and phase-wise dosimetric values (mean ± standard deviation over the 10 respiratory phases) for each patient and planning strategy. Bold values indicate mean values exceeding the corresponding clinical constraint.

Patient	Plan	$V_{20Gy}^{Lung-GTV}$ (%)	$D_{mean}^{Lung-GTV}$ (Gy)	$V_{22.5Gy}^{Heart}$ (%)	D_{mean}^{Heart} (Gy)	$D_{0.001cc}^{SC}$ (Gy)
Clinical constraint		< 30	< 19	< 50	< 10	< 45
Patient 1	$plan_{RL}$	5.53 ± 0.04	2.92 ± 0.02	0.00 ± 0.00	< 0.01	$43.65 \pm \mathbf{2.71}$
	$plan_{GTV}$	3.54 ± 0.10	1.96 ± 0.06	0.00 ± 0.00	< 0.01	13.82 ± 1.68
	$plan_{ITV}$	5.85 ± 0.07	3.35 ± 0.03	0.00 ± 0.00	0.01 ± 0.00	26.35 ± 5.31
Patient 2	$plan_{RL}$	6.41 ± 0.01	2.99 ± 0.01	0.59 ± 0.04	0.39 ± 0.02	0.05 ± 0.00
	$plan_{GTV}$	5.10 ± 0.11	2.93 ± 0.07	0.14 ± 0.05	0.16 ± 0.02	0.05 ± 0.00
	$plan_{ITV}$	12.59 ± 0.34	7.11 ± 0.19	1.20 ± 0.13	0.75 ± 0.07	0.06 ± 0.01
Patient 3	$plan_{RL}$	$\mathbf{30.46 \pm 0.11}$	16.99 ± 0.35	0.44 ± 0.29	0.48 ± 0.16	6.22 ± 3.55
	$plan_{GTV}$	27.22 ± 0.76	16.48 ± 0.57	1.49 ± 0.06	0.98 ± 0.02	0.94 ± 0.25
	$plan_{ITV}$	$\mathbf{33.00 \pm 0.53}$	$\mathbf{20.13 \pm 0.53}$	3.17 ± 0.12	1.89 ± 0.08	1.37 ± 0.55

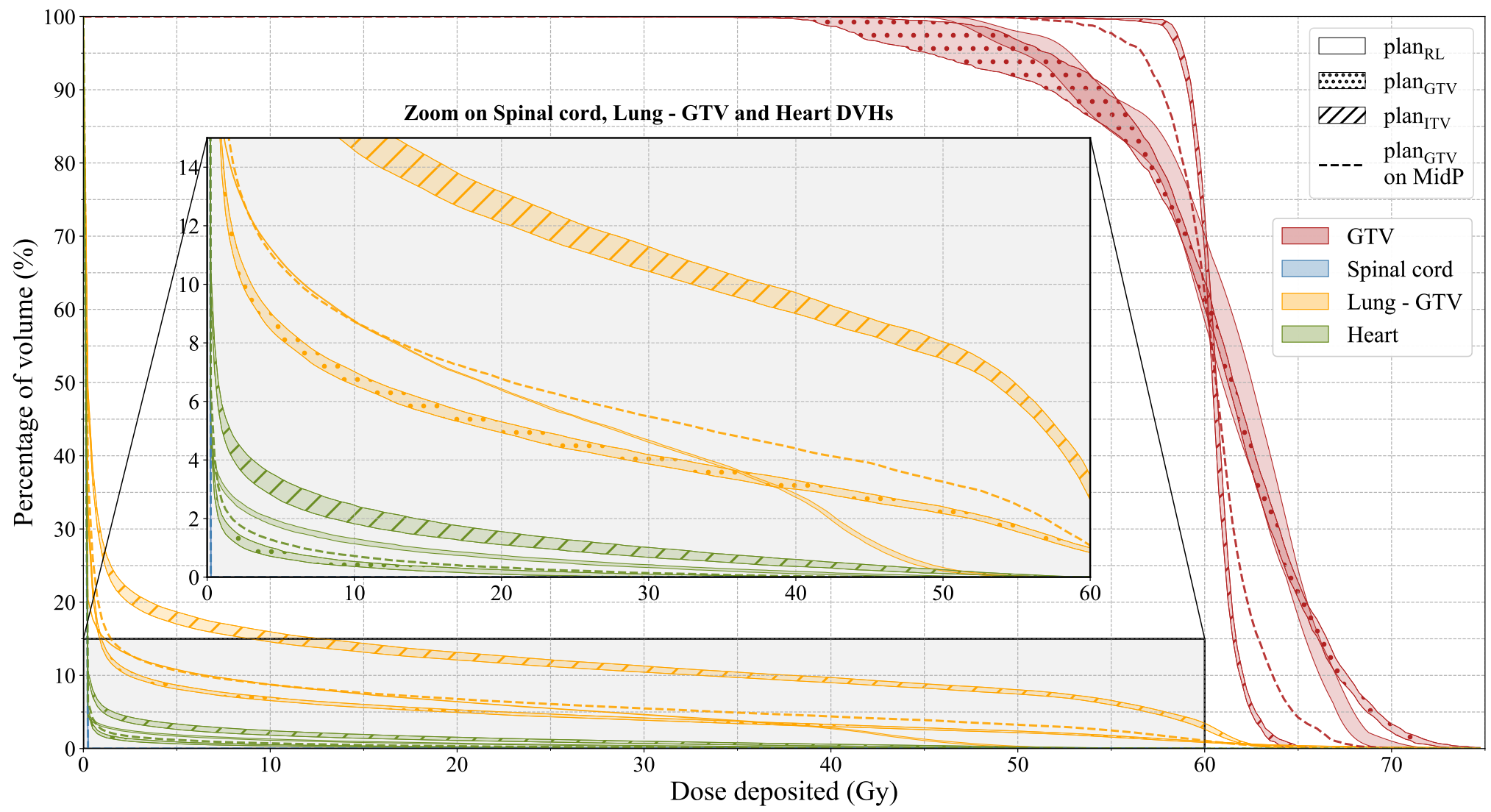

(a) Patient 2

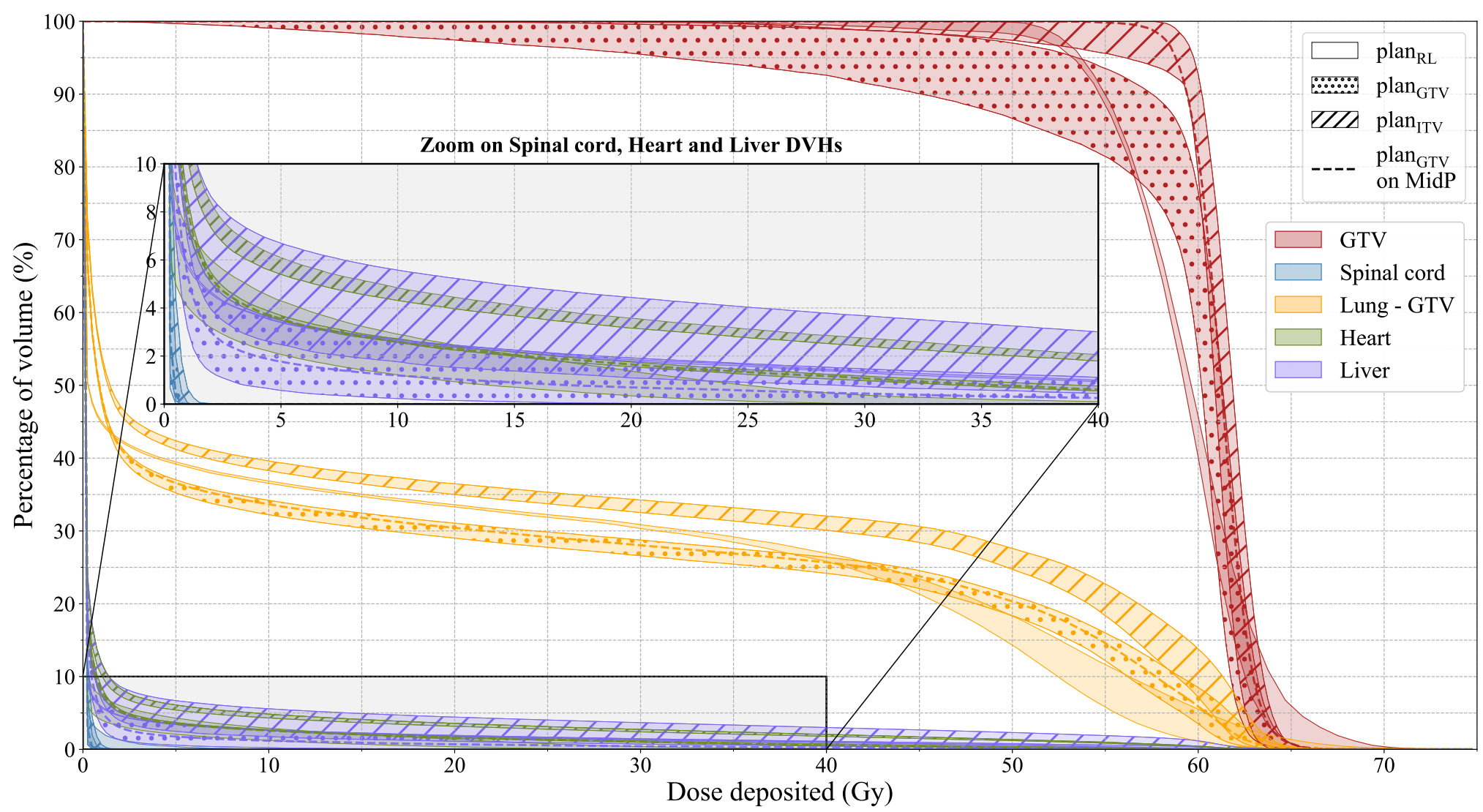

(b) Patient 3

Figure S3: DVH bands of the GTV and OAR for plan_{GTV} (in dotted areas), plan_{ITV} (in dashed zone) and plan_{RL} for (a) Patient 2 and (b) Patient 3. Bands denote the phase-wise range over the 10 respiratory phases of the 4DCT. The dashed line corresponds to plan_{GTV} delivered on the MidP CT. Results after the whole treatment delivery.
